# Supplementary material for: Time-dependent predictors of loss to follow-up in HIV care in low-resource settings: A competing risks approach
Source: PLoS One. 2025 Jul 24;20(7):e0329132. doi: 10.1371/journal.pone.0329132 (PMC12289015; doi:10.1371/journal.pone.0329132)
Supplement: S1 File — S1 Table. Univariate CHR and SHR analyses to identify predictors of LTFU among HIV-positive adults in Ethiopia (2019–2024). Legends: Abbreviations: CHR = cause-specific hazard ratio, SHR = subdistribution hazard ratio, CI = confidence interval, LTFU = loss to follow-up, Ref = reference, TPT = TB prevention therapy, TO = transfer out, WHO = World Health Organization. S2 Table. Fine and Gray SHR models stratified by follow-up period to assess time-varying effects on LTFU risk among HIV-positive adults, Ethiopia (2019–2024). Legends: Abbreviations: aSHR = Adjusted Sub-distribution Hazard Ratio, CI = Confidence interval, LTFU = Loss to Follow-Up, Ref = Reference, TPT = TB prevention Therapy; TO = Transfer Out, WHO = World Health Organization, * = P-value < 0.05, ** = P-value <0.01, *** = P-value <0.001. S3 Data. dta: De-identified dataset used for analysis. (ZIP) [file pone.0329132.s001.zip › Supporting Information file/S1 Table.docx]

**Supporting information (S1 Table)**

S1 Table presents the univariate cause-specific hazard ratio and subdistribution hazard ratio regression analyses used to identify candidate predictors of LTFU.

| **Covariates** | **LTFU (N=1116)** | **TO (N=534)** | **Death (N=349)** | **Unadjusted**  **CHR (95% CI)** | **P-value** | **Unadjusted**  **SHR (95% CI)** | **P-value** |
| --- | --- | --- | --- | --- | --- | --- | --- |
| **Age category** |  |  |  |  |  |  |  |
| 15-24 | 99 | 45 | 16 | 1.44 (1.14, 1.81) | 0.002 | 1.49 (1.18, 1.88) | 0.001 |
| 25-34 | 399 | 189 | 53 | 1.73 (1.48, 2.02) | <0.001 | 1.73 (1.48, 2.01) | <0.001 |
| 35-44 | 353 | 167 | 130 | 1.22(1.04, 1.43) | 0.014 | 1.23 (1.05, 1.44) | 0.010 |
| 45+ | 265 | 133 | 150 | Ref |  | Ref |  |
| **Sex** |  |  |  |  |  |  |  |
| Male | 490 | 198 | 191 | 1.20 (1.07, 1.35) | 0.002 | 1.16 (1.04, 1.31) | 0.010 |
| Female | 626 | 336 | 158 | Ref |  | Ref |  |
| **Address information** |  |  |  |  |  |  |  |
| Green | 838 | 448 | 306 | Ref |  | Ref |  |
| Yellow | 278 | 86 | 43 | 2.22 (1.94, 2.54) | <0.001 | 2.11 (1.85, 2.41) | <0.001 |
| **TPT status** |  |  |  |  |  |  |  |
| Gold | 452 | 247 | 83 | Ref |  | Ref |  |
| Bronze/silver/ | 664 | 287 | 266 | 4.46 (3.95, 5.04) | <0.001 | 3.02 (2.68, 3.39) | <0.001 |
| **Adherence** |  |  |  |  |  |  |  |
| Good | 412 | 225 | 163 | Ref |  | Ref |  |
| Poor | 704 | 309 | 186 | 4.58 (4.05, 5.18) | <0.001 | 3.62 (3.21, 4.09) | <0.001 |
| **Nutrition status** |  |  |  |  |  |  |  |
| Normal | 328 | 204 | 128 | Ref |  | Ref |  |
| Under-nourished | 683 | 270 | 172 | 2.79 (2.44, 3.19) |  | 2.50 (2.19, 2.85) | <0.001 |
| Overweight | 105 | 60 | 49 | 0.64 (0.51, 0.88) |  | 0.67 (0.54, 0.84) | <0.001 |
| **WHO clinical Stage** |  |  |  |  |  |  |  |
| I/II | 612 | 110 | 65 | Ref |  | Ref |  |
| III/IV | 504 | 424 | 284 | 1.10 (0.98, 1.24) |  | 0.89 (0.79, 0.99) | 0.048 |
