# Supplementary material for: Time-dependent predictors of loss to follow-up in HIV care in low-resource settings: A competing risks approach
Source: PLoS One. 2025 Jul 24;20(7):e0329132. doi: 10.1371/journal.pone.0329132 (PMC12289015; doi:10.1371/journal.pone.0329132)
Supplement: S1 File — S1 Table. Univariate CHR and SHR analyses to identify predictors of LTFU among HIV-positive adults in Ethiopia (2019–2024). Legends: Abbreviations: CHR = cause-specific hazard ratio, SHR = subdistribution hazard ratio, CI = confidence interval, LTFU = loss to follow-up, Ref = reference, TPT = TB prevention therapy, TO = transfer out, WHO = World Health Organization. S2 Table. Fine and Gray SHR models stratified by follow-up period to assess time-varying effects on LTFU risk among HIV-positive adults, Ethiopia (2019–2024). Legends: Abbreviations: aSHR = Adjusted Sub-distribution Hazard Ratio, CI = Confidence interval, LTFU = Loss to Follow-Up, Ref = Reference, TPT = TB prevention Therapy; TO = Transfer Out, WHO = World Health Organization, * = P-value < 0.05, ** = P-value <0.01, *** = P-value <0.001. S3 Data. dta: De-identified dataset used for analysis. (ZIP) [file pone.0329132.s001.zip › Supporting Information file/S2 Table.docx]

**Supporting information (S2 Table)**

The S2 Table presents results from Fine and Gray models stratified by follow-up period (0–12, 13–36, and 37–60 months) to supplement the understanding of time-varying relationships between covariates and the risk of LTFU within a competing risks framework

| **Covariates** | **0-12 months** | **13-36 months** | **37–60 months** |
| --- | --- | --- | --- |
|  | aSHR (95% CI) | aSHR (95% CI) | aSHR (95% CI) |
| **Age category** |  |  |  |
| 15-24 | 1.77 (1.35, 2.32)*** | 1.03(0.64, 1.65) | 1.34 (0.67, 2.64) |
| 25-34 | 1.52(1.24, 1.86)*** | 1.41(1.04, 1.89)* | 2.85(1.76, 4.62)** |
| 35-44 | 1.36(1.11, 1.65)** | 1.13(0.85, 1.52) | 1.16(0.71, 1.89) |
| 45+ | Ref | Ref | Ref |
| **Sex** |  |  |  |
| Male | 1.17(1.01, 1.36)* | 1.02(0.80, 1.31) | 1.47(1.01, 2.13)* |
| Female | Ref | Ref | Ref |
| **Address information** |  |  |  |
| Green | Ref | Ref | Ref |
| Yellow | 1.72(1.46, 2.02)*** | 1.35(1.01, 1.81)* | 1.61(1.03, 2.53 )* |
| **TPT status** |  |  |  |
| Gold | Ref | Ref | Ref |
| Bronze/silver/ | 1.19(1.02, 1.39)* | 1.78(1.39, 2.27)*** | 1.47(1.03, 2.11)* |
| **Adherence** |  |  |  |
| Good | Ref | Ref | Ref |
| Poor | 1.46(1.25, 1.69)*** | 2.89(2.27, 3.69)*** | 4.27(2.98, 6.12)*** |
| **Nutritional status** |  |  |  |
| Normal | Ref | Ref | Ref |
| Undernureshed | 1.57(1.33, 1.84)*** | 2.71(2.06, 3.56)*** | 2.46(1.57, 3.86)*** |
| Overweight | 0.83(0.62, 1.11) | 0.95(0.63, 1.42) | 0.96(0.56, 1.66) |
| **WHO clinical stage** |  |  |  |
| I/II | Ref | Ref | Ref |
| III/IV | 0.39(0.34, 0.46)*** | 0.46(0.36, 0.59)*** | 0.42(0.28, 0.62)*** |
