# Supplementary material for: Time-dependent predictors of loss to follow-up in HIV care in low-resource settings: A competing risks approach
Source: PLoS One. 2025 Jul 24;20(7):e0329132. doi: 10.1371/journal.pone.0329132 (PMC12289015; doi:10.1371/journal.pone.0329132)
Supplement: Attachment — Submitted filename: Response to Reviewers.docx [file pone.0329132.s002.docx]

**Response to Reviewers**
**Subject:** Revised Manuscript Submission

PONE-D-25-16597

Time-dependent predictors of loss to follow-up in HIV care in a low-resource settings: A competing risks approach

Dear Dr. Hamufare Mugauri,

Thank you for the opportunity to revise our manuscript. We sincerely appreciate the time and thoughtful feedback provided by you (editors) and the reviewers.

We have carefully addressed all comments raised by the academic editor and reviewers, and we are submitting the following revised materials for your consideration:

- A detailed rebuttal letter addressing each point raised by the editor and reviewers. This document is uploaded as a separate file labeled “Response to Reviewers.”
- A marked-up version of the manuscript showing all changes made from the original submission. This file is labeled “Revised Manuscript with Track Changes.”
- A clean version of the revised manuscript without tracked changes, labeled “Manuscript.”
- Updated supporting information.

We hope the revised manuscript meets your expectations and we look forward to your further evaluation and feedback.

Sincerely,

Tamrat Endebu, PhD Candidate

**Point raised by the academic editor**

Journal requirements:

When submitting your revision, we need you to address these additional requirements.

1. Please ensure that your manuscript meets PLOS ONE's style requirements, including those for file naming. The PLOS ONE style templates can be found at

<https://journals.plos.org/plosone/s/file?id=wjVg/PLOSOne_formatting_sample_main_body.pdf> and

[https://journals.plos.org/plosone/s/file?id=ba62/PLOSOne_formatting_sample_title_authors_affiliations.pdf](https://track.editorialmanager.com/CL0/https:%2F%2Fjournals.plos.org%2Fplosone%2Fs%2Ffile%3Fid=ba62%2FPLOSOne_formatting_sample_title_authors_affiliations.pdf/1/010f01975e0b5276-9cf05b89-47a1-43a5-9164-fda763adbbf5-000000/FI2XqgCHt0qLWNsO1-OtgkR5laoLCR0S3E50Og6POIE=215).

**Response:** Thank you for your guidance. We have made efforts to ensure compliance with PLOS ONE’s style requirements, including the file naming conventions.

2. Please provide additional details regarding participant consent. In the ethics statement in the Methods and online submission information, please ensure that you have specified (1) whether consent was informed and (2) what type you obtained (for instance, written or verbal, and if verbal, how it was documented and witnessed). If your study included minors, state whether you obtained consent from parents or guardians. If the need for consent was waived by the ethics committee, please include this information.

If you are reporting a retrospective study of medical records or archived samples, please ensure that you have discussed whether all data were fully anonymized before you accessed them and/or whether the IRB or ethics committee waived the requirement for informed consent. If patients provided informed written consent to have data from their medical records used in research, please include this information.

**Response:** Thank you for your valuable comment. We have now provided additional details as “All data were fully anonymized prior to access, and the requirement for informed consent was waived by the ethics committee, as the study involved retrospective analysis of deidentified data. We strictly maintained confidentiality by using anonymized records solely for research purposes.” (Manuscript: Line 161-163).

3. We note that your Data Availability Statement is currently as follows: [All relevant data are within the manuscript and its supporting information files.]

Please confirm at this time whether or not your submission contains all raw data required to replicate the results of your study. Authors must share the “minimal data set” for their submission. PLOS defines the minimal data set to consist of the data required to replicate all study findings reported in the article, as well as related metadata and methods (<https://journals.plos.org/plosone/s/data-availability#loc-minimal-data-set-definition>).

For example, authors should submit the following data:

- The values behind the means, standard deviations and other measures reported;

- The values used to build graphs;

- The points extracted from images for analysis.

Authors do not need to submit their entire data set if only a portion of the data was used in the reported study.

If your submission does not contain these data, please either upload them as Supporting Information files or deposit them to a stable, public repository and provide us with the relevant URLs, DOIs, or accession numbers. For a list of recommended repositories, please see <https://journals.plos.org/plosone/s/recommended-repositories>.

If there are ethical or legal restrictions on sharing a de-identified data set, please explain them in detail (e.g., data contain potentially sensitive information, data are owned by a third-party organization, etc.) and who has imposed them (e.g., an ethics committee). Please also provide contact information for a data access committee, ethics committee, or other institutional body to which data requests may be sent. If data are owned by a third party, please indicate how others may request data access.

**Response:** Thank you for your guidance. We confirm that we have included the minimal data set required to replicate the study findings. The de-identified dataset used for analysis has been uploaded as a Supporting Information file (S3 Data.dta). These materials provide all values used to compute the reported summary statistics, regression outputs, and figures.

[Note: HTML markup is below. Please do not edit.]

Reviewers' comments:

Reviewer's Responses to Questions

**Comments to the Author**

1. Is the manuscript technically sound, and do the data support the conclusions?

The manuscript must describe a technically sound piece of scientific research with data that supports the conclusions. Experiments must have been conducted rigorously, with appropriate controls, replication, and sample sizes. The conclusions must be drawn appropriately based on the data presented.

Reviewer #1: Yes

Reviewer #2: Yes

2. Has the statistical analysis been performed appropriately and rigorously?

Reviewer #1: Yes

Reviewer #2: Yes

3. Have the authors made all data underlying the findings in their manuscript fully available?

The [PLOS Data policy](http://track.editorialmanager.com/CL0/http:%2F%2Fwww.plosone.org%2Fstatic%2Fpolicies.action%23sharing/1/010f01975e0b5276-9cf05b89-47a1-43a5-9164-fda763adbbf5-000000/WfnYqsSc52eZ1IO5IrG0NiQuYCqPYjfS4lf3XbmtJWo=215) requires authors to make all data underlying the findings described in their manuscript fully available without restriction, with rare exception (please refer to the Data Availability Statement in the manuscript PDF file). The data should be provided as part of the manuscript or its supporting information, or deposited to a public repository. For example, in addition to summary statistics, the data points behind means, medians and variance measures should be available. If there are restrictions on publicly sharing data—e.g. participant privacy or use of data from a third party—those must be specified.

Reviewer #1: Yes

Reviewer #2: Yes

4. Is the manuscript presented in an intelligible fashion and written in standard English?

PLOS ONE does not copyedit accepted manuscripts, so the language in submitted articles must be clear, correct, and unambiguous. Any typographical or grammatical errors should be corrected at revision, so please note any specific errors here.

Reviewer #1: Yes

Reviewer #2: Yes

5. Review Comments to the Author

Please use the space provided to explain your answers to the questions above. You may also include additional comments for the author, including concerns about dual publication, research ethics, or publication ethics. (Please upload your review as an attachment if it exceeds 20,000 characters)

**Point raised by the reviewers**

Reviewer #1: I have enjoyed reading your well written manuscript on time-dependent predictors of LTFU in Ethiopia. Please see a few comments for your consideration.
Data processing and analysis
1. Line 139 - Consider using plural for data. Data were....

**Response:** Thank you for your overall positive feedback and valuable comments. We have edited as “Data were” (Manuscript: Line 133).

Results
Characteristics of a cohort
2. You included DSD enrolment numbers. Being an important LTFU intervention, it is not clear why this variable was not included in the LTFU risk model.

**Response:** Thank you for your valuable observation. We agree that DSD models are central to HIV care strategies and are often designed to reduce LTFU. While DSD was originally introduced as an intervention to improve retention, emerging evidence suggests that the type of DSD model itself may influence patient outcomes differently. Some studies have reported that certain DSD models, particularly those with shorter refill intervals (e.g., 3MMD), may not offer the same retention benefits as longer-term models like ASM/6MMD. Moreover, patients not enrolled in any DSD model may face structural or clinical barriers that place them at higher risk of disengagement from care. Given this, we considered DSD enrollment status a factor of LTFU and incorporated it into our risk models.

3. Up to 47% of patients were categorised in WHO Stage III and IV. This is a much higher proportion than would be expected from a mature HIV program. What is the reason for this, especially since these would be likely categorised as Advanced HIV Disease with enhanced interventions including those targeting LTFU.

**Response:** Thank you for your insightful comment. We acknowledge that the proportion of patients categorized as WHO Stage III/IV appears higher than expected in the context of a mature HIV program. However, several factors may explain this observation in our study. First, the dataset includes patients who initiated ART over the past five years, and for many, clinical staging was assessed at or near ART initiation, a period during which individuals commonly present with more advanced disease. This is particularly true in low-resource settings where delayed diagnosis and late initiation of ART remain challenges. Second, for patients retained in care, WHO clinical stage was recorded at the time of last contact. In contrast, for those who were LTFU (27%), died (8.4%), or transferred out (12.9%), WHO staging data were primarily recorded at the time of last contact or enrollment, with only a few having updates at their last contact. We have clarified this in the Methods section of the manuscript (Manuscript: Line 110-112). Given that LTFU, transfer out, and death often occur during the early months of ART, when many patients still present with advanced disease, this likely contributed to the higher proportion of Stage III/IV classifications observed in our cohort.

4. Age categories - The age categories analysed stop at 45yrs and above. It is not clear why older age categories were grouped together, as opposed to maintaining the age bands or at least to 50yrs and above. See some examples in published literature (<https://doi.org/10.1186/s12879-022-07505-0>; <https://doi.org/10.1186/s12889-020-8426-1>).

**Response:** Thank you for your insightful comment regarding the age categorization. We acknowledge that more granular age groupings, such as 45–49 and ≥50 years, are frequently used in the literature and can provide additional nuance. In our analysis, we categorized age into 15–24, 25–34, 35–44, and ≥45 years to maintain adequate statistical power, particularly for stratified analyses, where further disaggregation would have resulted in small cell sizes and potentially unstable estimates. We agree that future studies with larger sample sizes would benefit from employing more detailed age stratifications to enhance interpretability and comparability.

5. Correct the spelling of under-nourished in Table 4.

**Response:** Thank you for your observation. We have corrected the spelling to “undernourished” in Table 4.
6. The protective effect of WHO Stage III/IV on LTFU is an outlier. Would this be the same if you looked at each WHO Stage as a variable? See the shared examples above.

**Response:** Thank you for your valuable comment and thoughtful suggestion. We had already noted and discussed the unexpected protective association between advanced WHO clinical stages (III/IV) and LTFU in our initial analysis. As stated in the discussion section, our findings revealed that patients in WHO Stage III/IV had a significantly lower risk of LTFU compared to those in Stages I/II. This finding aligns with some previous studies while also contradicts with other several studies (Manuscript: Line 352-362). Based on your helpful suggestion, we re-analyzed the data using each WHO stage as a separate variable. However, the pattern remained largely consistent, with lower LTFU risk among patients in advanced stages. This finding may reflect context-specific programmatic factors and warrants further investigation.

Discussion
7. Line 266-269 and line 283-285. Results are repeated in the discussion section. I suggest that since these are already mentioned elsewhere, to only limit to what would be key for comparing and contrasting with published literature.

**Response:** Thank you very much for your insightful observation and helpful suggestion. We have revised the discussion section by removing the repeated results and focused instead on comparing our findings with existing literature (Manuscript: Lines 263–265 and 279–280).

**Reviewer #2**

Reviewer #2: Given that LTFU poses a serious threat to treatment outcomes, particularly viral suppression and the prevention of disease progression, this work is relevant. I commend the authors for their efforts and would like to offer the following suggestions:

1. The definition of LTFU used in the manuscript appears to omit a key conceptual component. LTFU, by standard practice, excludes individuals with documented outcomes such as confirmed death, formal transfer to another facility, or medical discontinuation of ART. These are not considered competing events but rather exclusions from the LTFU classification itself.
It is important to check the records of patients labeled as LTFU. Some may have died or transferred without documentation. Identifying these cases helps to avoid misclassification and improves competing risk analysis. I suggest that the authors clarify this distinction and align their operational definition accordingly.

**Response:** Thank you for your valuable comment and for highlighting this important conceptual distinction. We fully agree that, by standard practice, LTFU excludes individuals with documented outcomes such as confirmed death, formal transfer out, or medical discontinuation of ART. In our analysis, we adhered to this convention: only patients without any formal documentation of transfer, death, or treatment discontinuation, and who had interrupted their scheduled visit by at least 28 days, were classified as LTFU. As the reviewer correctly points out, some individuals recorded as LTFU may, in fact, have experienced undocumented deaths or unrecorded transfers. We have now clarified the operational definition of LTFU in the Methods section (Lines 90–93). In addition, while our use of a competing risks framework helps to mitigate potential bias from misclassification by modeling death and transfer as competing events, we acknowledge that imperfect data capture remains a limitation in retrospective routine datasets (Manuscript: Lines 364–367).

2. The current operational definition of LTFU in the study could lead to an overestimation of its incidence. In many HIV treatment programs, patients are dispensed ART for multiple months (e.g., 90 days) and are not necessarily required to return for a clinic visit during this period unless clinically indicated. Though some clinicians might schedule interim assessments for monitoring purposes. If such a patient misses an interim appointment but completes their medication course within the dispensation period, classifying them as LTFU based solely on missed interim visits may result in misclassification. For this reason, most studies define LTFU in reference to the next scheduled medication refill, not just any missed appointment. I suggest that the authors revisit their LTFU definition and consider this operational nuance to improve accuracy.

**Response:** Thank you for your thoughtful comment regarding the operational definition of LTFU. We agree that defining LTFU solely based on missed clinic visits without accounting for multi-month ART dispensing schedules may lead to overestimation. In response, we have revised our definition to reflect missed scheduled medication refill or clinic appointments by ≥28 consecutive days from the expected date, referencing the next scheduled refill wherever documented. For cases without explicitly recorded refill dates, we assumed standard 1–3 month follow-up intervals based on national guidelines. This clarification has now been included in the revised Methods section (Manuscript: Lines 93-98).

3. It would be helpful if the authors could specify the nature of the cohort used in the study. Was this an open cohort (allowing for rolling enrollment) or a closed cohort (fixed enrollment period)? Clarifying this will enhance the transparency of the methodology and allow for better interpretation of follow-up time and incidence rates.

**Response:** Thank you for your valuable observation and suggestion. We confirm that the study was based on an open cohort, allowing for rolling enrollment of HIV patients initiating ART over the study period. This design enabled inclusion of patients as they became eligible, thereby reflecting real-world clinical practice and enhancing the generalizability of our findings. We have now clarified this point in the Methods section (Manuscript: Lines 74 and 84-85).

4. For improved clarity and to aid in interpretation, I recommend that the authors present the results of the univariate analysis (CHR and SHR) prior to the adjusted multivariate models. This will allow readers to better understand the effect of covariates before and after adjustment.

**Response:** Thank you for your insightful suggestion regarding the presentation of unadjusted results. In response, we conducted univariate analyses using both the CHR and the SHR models to estimate the crude effects of each covariate on the risk of LTFU. These unadjusted results are now included in supporting information as “S1 Table” and a corresponding discussion has been added in the Results section (Manuscript: Lines 210–212).

6. PLOS authors have the option to publish the peer review history of their article ([what does this mean?](https://track.editorialmanager.com/CL0/https:%2F%2Fjournals.plos.org%2Fplosone%2Fs%2Feditorial-and-peer-review-process%23loc-peer-review-history/1/010f01975e0b5276-9cf05b89-47a1-43a5-9164-fda763adbbf5-000000/TlAIDs3frv-pA-KshzgVulQm3rSeQbmpqClb0F1988E=215)). If published, this will include your full peer review and any attached files.

If you choose “no”, your identity will remain anonymous but your review may still be made public.


**Do you want your identity to be public for this peer review?** For information about this choice, including consent withdrawal, please see our [Privacy Policy](https://track.editorialmanager.com/CL0/https:%2F%2Fwww.plos.org%2Fprivacy-policy/1/010f01975e0b5276-9cf05b89-47a1-43a5-9164-fda763adbbf5-000000/cAoN3lIplz2f5Wx4a7CY9hKKpL89WeEFHYd0lqkY09g=215).

Reviewer #1: **Yes:** Paul Wekesa

Reviewer #2: No

[NOTE: If reviewer comments were submitted as an attachment file, they will be attached to this email and accessible via the submission site. Please log into your account, locate the manuscript record, and check for the action link "View Attachments". If this link does not appear, there are no attachment files.]

While revising your submission, please upload your figure files to the Preflight Analysis and Conversion Engine (PACE) digital diagnostic tool, [https://pacev2.apexcovantage.com/](https://track.editorialmanager.com/CL0/https:%2F%2Fpacev2.apexcovantage.com%2F/1/010f01975e0b5276-9cf05b89-47a1-43a5-9164-fda763adbbf5-000000/-h9c0c6XHgfdla_gFRiqRmZOgbwdfqdN9McuARscUh4=215). PACE helps ensure that figures meet PLOS requirements. To use PACE, you must first register as a user. Registration is free. Then, login and navigate to the UPLOAD tab, where you will find detailed instructions on how to use the tool. If you encounter any issues or have any questions when using PACE, please email PLOS at [figures@plos.org](mailto:figures@plos.org). Please note that Supporting Information files do not need this step.

**Response:** Thank you. We have already uploaded our figure files to the Preflight Analysis and Conversion Engine (PACE) digital diagnostic tool to ensure compliance with PLOS formatting requirements.

 We look forward to your feedback and are happy to address any further comments.

Sincerely!

Tamrat Endebu
